# The Effect of Pain on Intolerance of Uncertainty and Death Anxiety in Individuals Developing Vertebral Fractures After Liver Transplantation

**DOI:** 10.3390/jcm15145634

**Published:** 2026-07-17

**Authors:** Ramazan Pasahan, Sami Akbulut, Funda Kavak Budak

**Affiliations:** 1 Department of Neurosurgery, Faculty of Medicine, Inonu University, 44280 Malatya, Turkey; r.pasahan@hotmail.com; 2 Department of Surgery and Liver Transplantation, Faculty of Medicine, Inonu University, 44280 Malatya, Turkey; akbulutsami@gmail.com; 3 Department of Psychiatric Nursing, Faculty of Nursing, Inonu University, 44280 Malatya, Turkey

**Keywords:** liver transplantation, vertebral fracture, pain, intolerance of uncertainty, death anxiety, patient

## Abstract

**Objective:** The aim of this study is to compare the levels of intolerance of uncertainty and death anxiety between individuals who developed vertebral fractures after liver transplantation and those who did not and to examine the effect of pain on these two variables. **Materials and Methods:** This study was designed as an observational cross-sectional study. Clinical and demographic data were retrospectively obtained from electronic medical records, while missing information and patient-reported outcomes (pain, intolerance of uncertainty, and death anxiety) were collected during a single structured telephone interview. The retrospective investigation was carried out among liver transplant recipients at İnönü University Turgut Özal Medical Center, comparing patients who developed vertebral fractures post-transplant with those who did not. Data were collected from the electronic medical records system of the hospital. Based on the evaluations obtained from the registration system, individuals diagnosed with vertebral fractures and individuals without vertebral fractures but with similar demographic characteristics (such as age, gender, duration of transplantation, and etiological factors) were included in the study. The data collection tools used were the “Patient Information Form,” “Visual Analog Scale (VAS),” “Intolerance of Uncertainty Scale,” and “Death Anxiety Scale.” The study employed Mann–Whitney U analysis, Spearman correlation analysis, and Fisher’s exact chi-square analysis. **Results:** Comparative analysis revealed statistically significant differences (*p* < 0.001) between post-liver transplant patients with vertebral fractures versus those without fractures across three key measures: pain intensity (VAS scores), intolerance of uncertainty, and death anxiety levels. A strong positive correlation was found between the patients’ pain levels and their level of intolerance of uncertainty (r = 0.823), as well as between pain levels and death anxiety (r = 0.840) (*p* < 0.001). Additionally, a strong positive correlation was identified between intolerance of uncertainty and death anxiety (r = 0.883) (*p* < 0.001). **Conclusions:** It was found that individuals with vertebral fractures had higher levels of pain, intolerance of uncertainty, and death anxiety compared to the group without vertebral fractures. As patients’ pain levels increased, their levels of intolerance of uncertainty and death anxiety also increased. Additionally, it can be stated that as patients’ intolerance of uncertainty increased, their death anxiety also increased.

## 1. Introduction

Liver transplantation is an effective treatment method preferred to extend life expectancy in cases of end-stage liver failure and certain liver diseases. While primarily addressing physical health, liver transplantation also profoundly impacts individuals socially, emotionally, and psychologically. Post-transplant complications—including steroid/immunosuppressant therapies, medication side effects, and pain—significantly affect both physical and mental well-being [1,2]. Vertebral fractures may occur in liver transplant patients as a result of osteoporosis caused particularly by immunosuppressive treatments and stress (through the effects of stress on the endocrine and immune systems) [3,4]. The likelihood of vertebral fractures within the first two years after liver transplantation is between 15 and 17% [5]. Vertebral fractures can negatively affect the physical functioning and psychological well-being of individuals after liver transplantation [5,6].

Studies have reported that vertebral fractures following liver transplantation cause severe physical pain in individuals, which disrupts patients’ quality of life [5,7]. In one study, it was found that anxiety and stress-related processes may influence neuroimmune mechanisms and contribute to altered pain perception [4]. Pain is a significant symptom that complicates daily life for these individuals and affects their adherence to treatment. Especially in cases of chronic pain, not only the physical condition but also the psychological stability of the individual can be deeply affected [8]. The persistence and unpredictability of pain may lead to the development of intolerance of uncertainty in individuals. Intolerance of uncertainty is a cognitive predisposition characterized by an individual’s excessive anxiety in the face of potential future threats and the development of negative cognitive interpretations regarding such situations [9]. Health-related uncertainties, in particular, are commonly observed in individuals during the post-transplantation period and can lead to an increase in psychological problems [9,10]. Studies have shown that increased intolerance of uncertainty plays a significant role in the rise in anxiety levels [11,12]. Complications, painful conditions, and uncertainties following transplantation may especially trigger thoughts related to death and increase death anxiety [13]. Death anxiety emerges as a significant psychological concern among liver transplant recipients. While transplantation symbolizes a renewed chance at life, it also inevitably forces patients to confront their mortality. Research demonstrates that post-transplant complications, medication side effects, and challenges in adapting to altered life circumstances and social roles collectively elevate anxiety levels in these patients [14,15,16]. This study aims to shed light on the psychological effects of chronic complications that develop after transplantation and to emphasize that pain management is important not only for physical outcomes but also for psychological well-being. The findings are expected to contribute to the development of a holistic care approach for individuals after transplantation.

Although previous studies have examined psychological problems, anxiety, and quality of life in liver transplant recipients, the psychological consequences of specific post-transplant complications, such as vertebral fractures, remain insufficiently explored. Existing literature has primarily focused on the physical consequences of vertebral fractures, including bone loss, fracture incidence, and pain; however, little is known about how these complications influence psychological processes such as intolerance of uncertainty and death anxiety. Vertebral fractures may represent not only a painful musculoskeletal complication but also a significant psychological stressor by increasing concerns about physical vulnerability, future health status, and mortality. Therefore, investigating the relationship between pain, intolerance of un-certainty, and death anxiety in liver transplant recipients with vertebral fractures may provide a more comprehensive understanding of the psychological burden associated with post-transplant complications. This perspective may contribute to the development of multidisciplinary care strategies addressing both physical and psychological needs after liver transplantation.

The aim of this study is to compare the levels of intolerance of uncertainty and death anxiety between individuals who developed vertebral fractures after liver transplantation and those who did not, and to examine the effect of pain on these two variables.

### Research Questions

- What are the levels of intolerance of uncertainty in individuals who developed vertebral fractures compared to those who did not after liver transplantation?
- What are the levels of death anxiety in individuals who developed vertebral fractures compared to those who did not after liver transplantation?
- What is the effect of pain on intolerance of uncertainty and death anxiety in individuals with and without vertebral fractures following liver transplantation?

## 2. Materials and Methods

### 2.1. Study Design and Setting

This was an observational cross-sectional study using retrospectively extracted clinical data from electronic medical records and prospectively collected patient-reported outcomes obtained through a single structured telephone interview. All patient-reported outcomes were assessed once during the study period.

### 2.2. Study Population

The study included liver transplant recipients who underwent transplantation at the Inönü University Liver Transplant Institute between September 2002 and January 2025, with and without post-transplant vertebral fractures. Patients without vertebral fractures were selected manually from the hospital registry and matched with patients with vertebral fractures according to age, sex, duration since transplantation, and transplantation etiology. Formal propensity score matching or algorithm-based matching was not performed.

### 2.3. Sample Size Calculation

Vertebral fractures were identified through radiological reports available in the electronic medical records. Patients were classified as having vertebral fractures if a diagnosis was confirmed by imaging findings and documented by the treating physicians. Sample size estimation was based on the findings of Paşahan et al. (2021) [5]. An a priori power analysis was performed with a 5% margin of error, 90% statistical power, and an estimated effect size of 0.65. The calculation indicated that a minimum of 36 participants (18 participants in each group) was required. Considering the possibility of incomplete data and potential participant loss, the target sample size was increased to 40 participants (20 participants in each group). During the study period, additional eligible participants were identified, and the final sample consisted of 50 participants, including 25 liver transplant recipients with vertebral fractures and 25 matched recipients without vertebral fractures. Vertebral fractures were identified through radiological reports available in the electronic medical records. Patients were classified as having vertebral fractures if the diagnosis was confirmed by imaging findings and documented by the treating physicians. During the study period, 25 liver transplant recipients with vertebral fractures met the eligibility criteria. No additional eligible patients with vertebral fractures were identified; therefore, no patients were excluded or declined participation. All eligible patients completed the structured telephone interview and were included in the final analysis.

### 2.4. Inclusion and Exclusion Criteria

Inclusion criteria: age ≥18 years, ≥6 months post-transplantation, and accessible medical records containing sufficient data. Exclusion criteria: severe cognitive impairment, diagnosed psychiatric disorders, or incomplete/invalid data.

### 2.5. Data Collection Procedure

Data were collected between March and June 2025. Clinical and demographic data were retrospectively extracted from the electronic patient records. Subsequently, each participant completed a single structured telephone interview lasting approximately 25 min, during which the study questionnaires (VAS, Intolerance of Uncertainty Scale–Short Form, and Death Anxiety Scale) were administered and any missing demographic information was obtained.

### 2.6. Data Collection Instruments

Patient Information Form: This form consists of eight items assessing age, sex, marital status, educational level, employment status, duration since transplantation, current medications, and comorbid chronic diseases other than liver transplantation.

Visual Analog Scale (VAS): The VAS is a methodologically robust, conceptually simple, and easy-to-administer tool suitable for pain assessment in surgical patients. It standardizes subjective pain reports into objective scores, facilitating consistent interpretation among healthcare providers. It is used to evaluate changes in pain severity following interventions or when pain-related issues arise [17]. Score interpretation: 0 = no pain, 1–2 = very mild pain, 3–4 = mild pain, 5–6 = moderate pain, 7–8 = severe pain, 9–10 = unbearable pain.

Intolerance of Uncertainty Scale–Short Form (IUS-12): IUS-12 developed by Carleton, Norton, and Asmundson (2007) [18], the original scale demonstrated high internal consistency (α = 0.94) and reliability (r = 0.74). The Turkish adaptation was conducted by Sarıçam et al. (2014) [19]. The 12-item scale comprises two subscales: prospective anxiety (items 1–7) and inhibitory anxiety (items 8–12). It is scored on a 5-point Likert scale, with total scores ranging from 12 to 60; higher scores indicate greater intolerance of uncertainty. No reverse-scored items are present. In this study, Cronbach’s alpha was 0.92.

Death Anxiety Scale (DAS): Developed by Templer (1970) [20] and validated in Turkey by Şenol (1989) [21], with further adaptation by Akça and Köse (2008) [22], this 15-item scale (true/false format) evaluates fear and anxiety related to one’s own death or the threat of death. Items 1–9 score “yes” = 1, “no” = 0; items 10–15 score “no” = 1, “yes” = 0. Total scores range from 0 to 15: 0–4 = mild, 5–9 = moderate, 10–14 = severe, and 15 = panic-level death anxiety [22]. In this study, Cronbach’s alpha was 0.94.

### 2.7. Statistical Analysis

SPSS version 20.0 (IBM Corp., Armonk, NY, USA) statistical software was used for data analysis. Descriptive characteristics of the participants were presented as frequencies and percentages. Descriptive statistics for the scores obtained from the scales and subscales were expressed as means and standard deviations. The normality of the distribution of the scales was assessed using skewness and kurtosis values. Since skewness and kurtosis values were not within the range of −1.5 to +1.5, the data were considered non-normally distributed. Differences in scale scores according to participants’ descriptive characteristics were evaluated using the Mann–Whitney U test, while categorical comparisons based on descriptive features were performed using Fisher’s exact chi-square analysis. In addition, Spearman’s correlation analysis was conducted to examine the relationship between mean scale scores. Statistical significance was set at *p* < 0.05, with a 95% confidence level. In addition to *p*-values, effect sizes were calculated for the Mann–Whitney U test results using the formula r = Z/√N. Effect sizes were interpreted according to conventional criteria, with values of 0.10, 0.30, and 0.50 representing small, moderate, and large effects, respectively. Missing demo-graphic and clinical information identified in electronic records was completed through structured telephone interviews. Participants with incomplete questionnaire responses were excluded from the analysis.

### 2.8. Study Protocol and Ethics Committee Approval

This observational cross-sectional study involving human participantshuman participants was in accordance with the ethical standards of institutional and national research committees and with the Helsinki Declaration of 1964 and its later amendments or comparable ethical standards. Ethical approval was ob-tained from the IRB of Inonu University for Non-Interventional Clinical Research (Approval no: 2025/8207).

## 3. Results

When comparing the descriptive characteristics of individuals with and without vertebral fractures following liver transplantation who participated in the study, no statistically significant differences were found, indicating a homogeneous distribution (*p* > 0.05, Table 1).

Comparison of pain, intolerance of uncertainty, and death anxiety scores between liver transplant recipients with and without vertebral fractures revealed statistically significant differences between the groups (*p* < 0.001). Another aspect that should be considered is the relatively advanced age of the patients with vertebral fractures and the proportion of patients who underwent liver transplantation because of malignant disease. Although no statistically significant differences were observed between the groups with respect to demographic and clinical characteristics, advanced age is a well-established risk factor for osteoporosis and vertebral fractures. In addition, patients transplanted for malignant disease may experience greater psychological distress due to concerns about disease recurrence, long-term prognosis, and mortality. These factors may have contributed to the higher levels of intolerance of uncertainty and death anxiety observed in the vertebral fracture group and should be taken into consideration when interpreting the present findings. Patients with vertebral fractures had higher pain scores (5.44 ± 0.86), intolerance of uncertainty scores (53.56 ± 2.63), and death anxiety scores (11.88 ± 1.71) compared with patients without vertebral fractures, who had lower scores for pain (3.32 ± 0.55), intolerance of uncertainty (40.52 ± 5.05), and death anxiety (7.92 ± 1.49). These results demonstrate that patients with vertebral fractures had significantly higher levels of pain, intolerance of uncertainty, and death anxiety compared with those without vertebral fractures (Table 2).

Spearman correlation analysis revealed strong positive correlations between pain scores and intolerance of uncertainty scores (r = 0.823, *p* < 0.001), pain scores and death anxiety scores (r = 0.840, *p* < 0.001), and intolerance of uncertainty scores and death anxiety scores (r = 0.883, *p* < 0.001). These findings indicate that higher pain scores were associated with higher levels of intolerance of uncertainty and death anxiety. Additionally, higher intolerance of uncertainty scores were associated with higher death anxiety scores (Table 3).

## 4. Discussion

The findings of this study, which examined the relationship between pain, intolerance of uncertainty, and death anxiety in individuals who developed vertebral fractures after liver transplantation, were evaluated in light of the literature. To the best of our knowledge, this is the first study to investigate the relationships among pain, intolerance of uncertainty, and death anxiety in liver transplant recipients with vertebral fractures. Therefore, direct comparisons with previous studies are limited. Where appropriate, our findings were interpreted in light of studies conducted in liv-er transplant recipients and other populations experiencing chronic pain or vertebral fractures.

It was determined that patients with vertebral fractures following liver trans-plantation had moderate levels of pain, high levels of intolerance of uncertainty, and severe levels of death anxiety. Previous studies have shown that vertebral fractures developing after liver transplantation increase pain levels in patients [5,7]. Additionally, the uncertainties experienced by patients related to the transplantation process and vertebral fractures may be associated with higher anxiety levels and consequent intolerance. In particular, vertebral fractures developing after a life-threatening process such as liver transplantation may adversely affect the individual’s body perception, functionality, and quality of life, potentially contributing to increased thoughts related to death. This, in turn, is expected to increase death anxiety. This study revealed that pain in this context is not merely a physical experience but also a psychological factor that reinforces anxiety related to the possibility of death. Intolerance of uncertainty is defined as the tendency to perceive uncertain or unpredictable situations as threatening and to exhibit low tolerance toward these situations. In individuals who have undergone serious health challenges such as liver transplantation and subsequent vertebral fractures, uncertainty about health status, the course of complications, or the possibility of recurrence can undermine the sense of control, creating a persistent feeling of threat. This perceived threat may be associated with increased death anxiety, which represents an existential form of psychological distress.

Patients without vertebral fractures following liver transplantation were found to have mild pain levels, moderate intolerance of uncertainty, and moderate death anxiety.

The longer duration since transplantation may partly explain the lower pain levels observed in this group. During the post-transplant period, factors such as organ rejection, initiation of immunosuppressive therapy, presence of infections, frequent medical check-ups, and complications related to the bile duct can contribute to moderate levels of intolerance of uncertainty and death anxiety in patients [6,23].

Studies have shown that liver transplant patients may experience increased anxiety due to feelings of guilt over potential complications in donors, undergoing immuno-suppressive treatment, and concerns about not regaining their previous physical condition [24,25]. Uncertainties regarding the challenges they may face during the post-transplant disease and treatment process play a significant role in increasing anxiety levels [6,26]. The findings of this study are consistent with the existing literature.

When comparing pain, intolerance of uncertainty, and death anxiety levels be-tween individuals with and without vertebral fractures after liver transplantation, a statistically significant difference was found (*p* < 0.05). It was determined that individuals with vertebral fractures had higher levels of pain, intolerance of uncertainty, and death anxiety compared to those without fractures. The higher pain levels in individuals with vertebral fractures can be attributed to the nature of bone fractures, which typically cause continuous or recurrent pain. This finding aligns with previous research indicating that chronic pain affects not only the physiological but also the psychological state of the individual [27]. The sense of loss of control and unpredictability caused by pain, along with the addition of a secondary illness to the existing condition, may explain the higher intolerance of uncertainty levels observed in individuals with vertebral fractures. Complications arising during and after the trans-plant process can make the reality of death more tangible for these individuals. Physically debilitating conditions such as vertebral fractures may increase awareness of the inevitability of death, and may be associated with higher levels of death anxiety compared with individuals without fractures.

Although the study groups were matched for age, sex, duration since transplantation, and transplantation etiology, several additional factors may have influenced the observed psychological outcomes. Advanced age, underlying malignant disease, coexisting chronic medical conditions, cumulative corticosteroid exposure, immuno-suppressive therapy, osteoporosis severity, functional limitations, and overall physical health may all contribute to pain perception, intolerance of uncertainty, and death anxiety. Therefore, the present findings should be interpreted within the context of these potential confounding factors. Future studies using larger samples and multivariable analytical approaches are warranted to clarify the independent contribution of vertebral fractures to psychological outcomes.

Higher pain levels were associated with higher intolerance of uncertainty and death anxiety scores. Additionally, it was determined that as the patients’ intolerance of uncertainty levels increased, their death anxiety levels also rose. These findings reveal that chronic pain affects not only physical but also psychological processes. Pain is thought to reduce the individual’s predictability of the future, thereby increasing the perception of uncertainty, which in turn elevates death anxiety. Particularly, as the intensity of pain increases, feelings of insecurity about the future, loss of control, and frequency of death-related thoughts become more pronounced in individuals.

Although pain was strongly associated with intolerance of uncertainty and death anxiety, these psychological outcomes are likely influenced by multiple additional factors. Liver transplant recipients may experience fear of graft rejection, concerns regarding lifelong immunosuppressive therapy, recurrent hospitalizations, reduced physical functioning following vertebral fractures, uncertainty about future health, financial burden, dependence on caregivers, and decreased quality of life. These factors may independently or collectively contribute to psychological distress and should be considered when interpreting the present findings.

Future studies should evaluate these variables using multivariable analyses. The present findings have important clinical implications. Routine assessment of pain alone may be insufficient in liver transplant recipients who develop vertebral fractures. Screening for intolerance of uncertainty and death anxiety may facilitate early psychological interventions, improve coping strategies, and potentially contribute to better quality of life outcomes.

## 5. Conclusions

In this study, it was found that individuals who developed vertebral fractures after liver transplantation had significantly higher levels of pain, intolerance of uncertainty, and death anxiety compared to those without fractures. Additionally, it was determined that as pain levels increased, intolerance of uncertainty and death anxiety also increased correspondingly. These results indicate that the development of vertebral fractures negatively affects not only the physical but also the psychological quality of life of liver transplant patients. Pain is an important factor that is often overlooked in chronic diseases but directly affects the psychological state of the individual. Intolerance of uncertainty, especially in life-threatening conditions such as liver transplantation where future anxieties are high, can reduce patients’ psychological well-being. The increase in death anxiety may negatively impact both the patient’s adherence to treatment and overall quality of life. It is important to consider the psychological effects of additional physical complications such as vertebral fractures and to provide psychosocial support to these individuals. Integrating intervention programs targeting psychological variables such as intolerance of uncertainty and death anxiety (e.g., psychoeducation, counseling, psychotherapy) alongside physiological treatments for pain (e.g., vertebroplasty, stabilization, algological procedures) into healthcare services will support the overall well-being of patients.

## 6. Limitations of the Study

This study has several limitations. First, due to its observational cross-sectional design, causal relationships between pain, intolerance of uncertainty, and death anxiety cannot be established. Second, patient-reported outcomes were assessed at a single time point, which may not reflect changes in psychological status over time. Third, some information was obtained through structured telephone interviews and therefore may be subject to recall bias and social desirability bias. In addition, conducting the study at a single transplantation center with a relatively small sample may limit the generalizability of the findings. Future multicenter studies with larger and more diverse populations, as well as longitudinal and interventional designs, are needed to better clarify these relationships and evaluate strategies for reducing pain, intolerance of uncertainty, and death anxiety. Additionally, potential confounding factors, including severity of osteoporosis, cumulative corticosteroid exposure, analgesic use, pre-transplant psychiatric history, and socioeconomic characteristics, could not be fully evaluated due to limitations in retrospective data availability. Future prospective studies incorporating these variables are recommended to better clarify the independent contribution of vertebral fractures and pain to psychological outcomes. Furthermore, although structured telephone interviews allowed the collection of patient-reported outcomes, they may have introduced recall bias because participants were required to report subjective experiences retrospectively. Potential confounding factors such as osteoporosis severity, corticosteroid exposure, analgesic use, previous psychiatric history, and socioeconomic status were not evaluated and may have influenced psychological outcomes. Furthermore, although patients were matched according to age, sex, duration since transplantation, and transplantation etiology, other potential confounding factors—including underlying malignant disease, osteoporosis severity, cumulative corticosteroid exposure, immunosuppressive treatment, analgesic use, functional status, comorbid chronic diseases, and socioeconomic characteristics—were not controlled for in the analyses and may have influenced the observed psychological outcomes. Future prospective studies incorporating multivariable analyses are recommended to better clarify the independent contribution of vertebral fractures and pain to intolerance of uncertainty and death anxiety.

## Figures and Tables

**Table 1:** Comparison of Patients According to Their Demographic Characteristics.

Descriptive Characteristics	Patients with Vertebral Fracture		Patients Without Vertebral Fracture		*p*-Value
Age	*n*	%	*n*	%	
60–70 years	9	36.0	7	28.0	0.083
71–81 years	5	20.0	5	20.0	
82 years and above	11	44.0	13	52.0	
Gender					
Female	12	48.0	11	44.0	0.248
Male	13	52.0	14	46.0	
Marital Status					
Married	25	100.0	25	100.0	
Single	0	0	0	0	
Education Level					
Literate	7	28.0	7	28.0	
Primary School	18	72.0	18	72.0	
Income Level					
Income less than expenses	0	0	0	0	
Income equals expenses	25	100.0	25	100.0	
Income more than expenses	0	0	0	0	
Employment Status					
Employed	0	0	0	0	
Unemployed	25	100.0	25	100.0	
Time Since Transplantation					
0–2 years	2	8.0	3	12.0	0.113
3–5 years	11	44.0	14	56.0	
6 years and above	12	48.0	8	32.0	
Presence of Other Chronic Diseases					
Yes	25	100.0	25	100.0	
No	0	0	0	0	
Used Immunosuppressive Drugs					
Tacrolimus	13	52.0	14	56.0	0.386
Mycophenolate mofeti	12	48.0	11	44.0	
Reason for Liver Transplantation					
Tumor	10	40.0	7	28.0	
Biliary Tract	7	28.0	8	32.0	0.412
Liver Failure	8	32.0	10	40.0	

Note: *p* < 0.05 is significant.

**Table 2:** Comparison of Pain, Intolerance of Uncertainty, and Death Anxiety Levels in Patients With and Without Vertebral Fracture.

Parameters	Vertebral Fracture Group (*n* = 25) Mean ± SD	No Vertebral Fracture Group (*n* = 25) Mean ± SD	Mean Rank	U	*p*	Effect Size (r)
Pain	5.44 ± 0.86	3.32 ± 0.55	37.46/13.54	13.500	˂0.001	082
Intolerance of uncertainty	53.56 ± 2.63	40.52 ± 5.05	37.70/13.22	5.500	˂0.001	0.84
Death anxiety	11.88 ± 1.71	7.92 ± 1.49	36.78/14.22	3.500	˂0.001	0.85

Note: SD = standard deviation; *p* < 0.05 was considered statistically significant.

**Table 3:** Correlation Analysis Results Between the Scales.

	Intolerance of Uncertainty	Death Anxiety
Pain	r = 0.823 *p* < 0.001	r = 0.840 *p* < 0.001
Intolerance of Uncertainty		r = 0.883 *p* < 0.001

r = correlation coefficient; *p* < 0.05 was considered statistically significant.

## Data Availability

The data used to support the findings of this study are available from the corresponding author upon request.
